# Self-Assembled Curcumin–Epigallocatechin Gallate Nanoparticles Enhance Antiviral Activity Against White Spot Syndrome Virus Through Immunomodulation, Antioxidant Effects, and Metabolic Regulation

**DOI:** 10.3390/antiox15101291

**Published:** 2026-10-05

**Authors:** Songlin Luo, Xudong Yang, Shunqiang Wei, Chushun Yi, Fansheng Zeng, Fali Zhang, Wei Zhang, Xiuli Chen, Yongzhen Zhao, Yinghui Wang, Aiguo Huang

**Affiliations:** 1 Guangxi Laboratory on the Study of Coral Reefs in the South China Sea, Coral Reef Research Center of China, School of Marine Sciences, Guangxi University, Nanning 530004, China; luosonglin0528@163.com (S.L.); 18577889437@163.com (S.W.); 13617876509@163.com (C.Y.); zfs2527300037@163.com (F.Z.); zhangwei20@gxu.edu.cn (W.Z.); 2 Institute of Green and Low Carbon Technology, Guangxi Institute of Industrial Technology, Nanning 530201, China; yangxudong@rhx-tech.com (X.Y.); dts_zfl@126.com (F.Z.); 3 Guangxi Key Laboratory of Aquatic Genetic Breeding and Healthy Aquaculture, Guangxi Academy of Fishery Sciences, Nanning 530021, China; chenxiuli2001@163.com (X.C.); fisher1152002@126.com (Y.Z.)

**Keywords:** white spot syndrome virus, *Procambarus clarkii*, self-assembled nanoparticles, antiviral activity, metabolic remodeling

## Abstract

White Spot Syndrome Virus (WSSV) remains a major factor limiting the sustainable development of crustacean aquaculture, creating an urgent need to develop comprehensive antiviral strategies with multiple protective functions. Natural bioactive compounds with antiviral, immunomodulatory, and antioxidant activities hold great potential for application; however, their poor stability and low bioavailability limit their practical use. In this study, self-assembled nanosystem (CE SA) composed of epigallocatechin gallate (EGCG) and curcumin (Cur) was constructed based on noncovalent interactions, and its protective effects against WSSV-infected *Procambarus clarkii* were systematically evaluated. The results indicate that CE SA exhibits good stability, sustained-release properties, and biosafety. Compared with Cur, EGCG, and their physical mixture (CE), CE SA significantly improved the survival rate of infected shrimp and reduced viral copy numbers as well as the expression levels of key viral genes such as *IE1*, *DNApol*, and *VP28*. At the same time, CE SA significantly promoted the expression of relevant genes such as *Dorsal*, *Relish*, *SOD*, and *CAT*, increased SOD and CAT enzyme activities and hematopoietic cell viability, and effectively alleviated damage to the hepatopancreas and intestinal tissues. Furthermore, metabolomic analysis indicated that CE SA regulates key metabolic processes such as amino acid metabolism, inflammation-related metabolism, nucleotide metabolism, and redox homeostasis, further elucidating its potential mechanism for enhancing the host’s antiviral capacity. In summary, by enhancing the stability and utilization efficiency of natural bioactive components, CE SA achieves superior anti-WSSV efficacy compared to individual components. Its protective effects are closely related to the inhibition of viral replication, enhancement of the immune response, improvement of antioxidant defenses, and regulation of host metabolic homeostasis. This study provides a promising, self-assembling nanostructure strategy for the green prevention and control of viral diseases in aquatic animals.

## 1. Introduction

White spot syndrome virus (WSSV) is currently one of the most devastating viral pathogens in the aquaculture industry, characterized by rapid transmission, a broad host range, and high mortality rates [1,2]. Since its emergence, WSSV has become widespread among major farmed shrimp species worldwide, causing significant economic losses, and is considered one of the key factors limiting the sustainable development of the crustacean aquaculture industry [3,4]. WSSV infection can disrupt the innate immune homeostasis of crustacean hosts, affecting the expression of immune signaling pathways such as Toll and IMD, as well as downstream immune factors. At the same time, viral infection can lead to excessive accumulation of reactive oxygen species (ROS), triggering oxidative stress and oxidative damage to cellular components such as lipids and proteins [5]. Although aquaculture management and biosecurity measures have been continuously improved, the lack of safe and effective antiviral interventions means that WSSV remains a major factor limiting industry development.

In recent years, natural bioactive compounds derived from plants have garnered widespread attention in the field of aquatic viral disease prevention and control due to their excellent biocompatibility and ability to regulate multiple targets [6,7]. However, most natural bioactive molecules generally suffer from poor water solubility, insufficient stability, susceptibility to degradation, and low bioavailability [8,9,10]. These issues make it difficult to maintain sustained effective concentrations in complex aquaculture environments and within living organisms, thereby limiting their practical application. Consequently, improving the stability, delivery efficiency, and sustained action of natural bioactive compounds has become a critical scientific challenge in advancing their industrial applications.

Self-assembling nanotechnology offers a new research approach to addressing the aforementioned issues. This strategy leverages noncovalent interactions—such as hydrogen bonding, π–π stacking, and hydrophobic interactions—between natural small molecules to spontaneously form nanostructures, enabling efficient assembly without the need for additional carrier materials [11]. Compared to traditional nanodelivery systems, self-assembly systems offer advantages such as simple preparation, high active ingredient content, good biosafety, and ease of scaling up. At the same time, they can enhance the stability of active ingredients, prolong their retention time in vivo, and enable sustained release [12]. Therefore, self-assembly technology is considered an important direction for improving the utilization efficiency of natural active compounds and promoting their practical applications.

Epigallocatechin gallate (EGCG), a major active component of tea polyphenols, possesses antioxidant, anti-inflammatory, and antiviral activities [13]. Studies have shown that EGCG exerts anti-WSSV effects by inhibiting viral replication, modulating immune responses, and alleviating oxidative stress [14,15]. However, EGCG is prone to oxidative degradation and has poor stability, which limits its duration of action and efficacy in vivo [16,17]. Curcumin (Cur) is another natural polyphenolic compound with excellent antioxidant, anti-inflammatory, and immunomodulatory properties [18,19]. Its molecular structure is rich in aromatic rings and phenolic hydroxyl groups, it can participate in nanostructure formation through hydrogen bonding and π–π stacking interactions [20,21]. Therefore, the synergistic assembly of Cur and EGCG is expected to enhance the stability and in vivo retention time of EGCG, thereby strengthening its antiviral effects. Given their excellent structural complementarity, Cur is not only expected to act as a bioactive component in regulating the host’s disease resistance but can also enhance the stability and bioavailability of EGCG through synergistic self-assembly, thereby boosting the overall antiviral efficacy [22,23]. However, research on the application of self-assembly systems using natural bioactive compounds in the prevention and control of aquatic animal diseases remains relatively limited, and their structure–activity relationships and antiviral advantages still need to be further elucidated.

Based on this, this study employed self-assembly strategy to construct a Cur-EGCG self-assembled nanosystem (CE SA) and systematically characterized its physicochemical properties, stability, and sustained-release performance. Furthermore, this study evaluated the protective effect of CE SA against WSSV infection, as well as its regulatory effects on the host immune response, antioxidant capacity, and metabolic homeostasis. By integrating analyses of viral replication levels, immune defense, oxidative stress, and non-targeted metabolomics, this study aims to elucidate the potential mechanisms by which self-assembly of natural active compounds enhances bioavailability and synergistic antiviral activity, thereby providing a theoretical basis and technical support for the application of natural product nanotechnology in the green prevention and control of viral diseases in aquatic animals.

## 2. Materials and Methods

### 2.1. Materials

Curcumin (C_21_H_20_O_6_), Epigallocatechin gallate (C_12_H_18_O_11_), Polyvinylpyrrolidone (C_6_H_9_NO)_n_, Dimethyl sulfoxide (C_2_H_6_OS), Sodium dodecyl sulfate (C_12_H_25_NaO_4_S), Phosphotungstic acid (H_3_PW_12_O_40_), Sodium citrate (Na_3_C_6_H_5_O_7_), Citric acid (C_6_H_8_O_7_), Phosphoric acid (H_3_PO_4_) and Acetonitrile (CH_3_CN) were purchased from Aladdin Reagents (Shanghai, China). Glucose (C_6_H_12_O_6_), Disodium hydrogen phosphate (Na_2_HPO_4_), Potassium dihydrogen phosphate (KH_2_PO_4_), Calcium chloride (CaCl_2_) and Manganese(II) chloride (MnCl_2_) were purchased from Macklin Reagents (Shanghai, China). Potassium bromide (KBr) and Sodium chloride (NaCl) were obtained from Cologne Chemicals (Chengdu, China). Leibovitz’s L-15 Medium. Anhydrous ethanol (CH_3_CH_2_OH), of analytical grade, was supplied by Sinopharm Chemical Reagent Co., Ltd (Shanghai, China). All water used in the experiments was deionized water (conductivity < 10^−6^ S/cm).

### 2.2. Synthesis of CE SA

Polyvinylpyrrolidone (PVP) was dissolved in water to obtain a 0.5 mg/mL solution. Cur and EGCG were then dissolved in dimethyl sulfoxide (DMSO) to obtain 10 mg/mL and 5 mg/mL solutions, respectively. Subsequently, 1 mL of each of the above solutions was added dropwise to the PVP solution and stirred at 25 °C and 500 rpm for 10 min, resulting in a final Cur concentration of 1 mg/mL and EGCG concentrations of 200, 400, 600, 800, and 1000 μg/mL. The product was dialyzed in water (MWCO 3500D) for 24 h to remove the organic solvent. Centrifuge at 4 °C and 8000 rpm for 10 min, then freeze-dry.

### 2.3. Characterization of CE SA

The structure and physicochemical properties of CE SA were systematically characterized. After applying a gold sputter coating to an appropriate amount of the sample and mounting it on a conductive substrate, the surface morphology was observed using a scanning electron microscope (SEM) (HITACHI, Tokyo, Japan). After depositing the sample onto a copper mesh and allowing it to air-dry, the microstructure was analyzed using a transmission electron microscope (TEM) (Hitachi High-Tech, Tokyo, Japan). After drying the samples, X-ray diffraction (XRD) (Rigaku, Tokyo, Japan) was performed to analyze their crystalline properties; simultaneously, Fourier-transform infrared spectroscopy (FTIR) (Thermo Fisher Scientific, Waltham, MA, USA) and ultraviolet–visible absorption spectroscopy (UV–vis) (Shimadzu, Kyoto, Japan) were used to characterize their intermolecular interactions and optical properties. Furthermore, the samples were prepared as suspensions, and their hydrodynamic particle size distribution was determined using a nanoparticle size and zeta potential analyzer (Malvern Instruments, Malvern City, UK). Surface charge characteristics and dispersion stability were analyzed via zeta potential measurements.

### 2.4. In Vitro pH Stability of CE SA

To evaluate the stability of CE SA particles under different pH conditions, samples were dispersed in buffer solutions of different pH levels (pH 4.0, 7.0, and 10.0) to prepare dispersions of specific concentrations. These were then incubated at room temperature in the dark, and samples were collected at predetermined time points (0, 0.5, 1, 2, 4, 8, 12, 24, 36, 48, and 72 h). The changes in the absorbance of their characteristic absorption peaks were measured using a UV spectrophotometer.

### 2.5. In Vitro Release Study by CE SA

To evaluate the in vitro release behavior of CE SA, the dialysis bag method was employed. A specific volume of CE SA dispersion was placed into a pretreated dialysis bag (MWCO 3500D), sealed, and placed in a release medium containing buffer solutions of different pH levels (pH 4.0, 7.0, and 10.0). Release experiments were conducted at room temperature under gentle agitation (25 °C, 100 rpm). During the release process, a specific volume of the external buffer solution was withdrawn at regular intervals (0, 0.5, 1, 2, 4, 8, 12, 24, 36, 48, and 72 h), and an equal volume of fresh buffer solution was added simultaneously. Add NaOH solution to the extracted solution; after color development, measure the absorbance at 450 nm using a microplate reader, and calculate the cumulative release at each time point based on the standard curve. This experiment was repeated three times.

### 2.6. Laboratory Animals and the Development of Infection Models

Healthy crayfish (approximately 15–20 g each) were purchased from a local farm in Nanning, Guangxi. The crayfish were reared in tanks for 7 days to acclimate them to the experimental environment. During this period, the water temperature was maintained at 25 ± 1 °C, and a partial water change was performed daily. Feeding was discontinued 24 h before the experiment, and crayfish were randomly selected for PCR testing to confirm they were free of WSSV. All animal experimentation protocols have been approved by the Guangxi University Committee on the Ethics of Laboratory Animal Welfare (GXU-2024-214).

To evaluate the therapeutic efficacy following viral infection and determine the optimal concentration of CE SA, the experiment was divided into a WSSV group (control group) and treatment groups: Cur/WSSV, EGCG/WSSV, Cur + EGCG (CE)/WSSV, and CE SA/WSSV (at doses of 25, 50, 100, and 200 mg/kg, respectively). The WSSV infection model was established via intraperitoneal injection: the control group received an injection of 1 × 10^7^ copies of a pre-prepared WSSV viral suspension into the second abdominal segment muscle of the crayfish, while the experimental groups received an injection of an equal volume of a mixture of the test sample and WSSV. Each group included three replicate samples, with five crayfish per replicate (*n* = 3 × 5). After injection, the crayfish were reared in standard isolated water bodies under identical environmental conditions, and samples were collected 24 h later for further analysis.

The study on the protective effects on crayfish was divided into the TNE group (negative control), the WSSV group (positive control), and the experimental groups (Cur/WSSV group, EGCG/WSSV group, CE/WSSV group, and CE SA/WSSV group). Survival rates were continuously monitored and recorded for each group for 7 days following injection. Each treatment group consisted of three replicates, with 20 crayfish in each replicate.

### 2.7. Safety Assessment

Healthy crayfish were randomly divided into a control group and an experimental group. The control group was injected with TNE buffer (10 mM Tris-HCl, 100 mM NaCl, 1 mM EDTA); in the experimental group, Cur, EGCG, and CE SA were diluted with TNE buffer to concentrations of 25, 50, 100, and 200 mg/kg, respectively, and injected using the same method as described above. Each group included three replicates, with five crayfish per replicate (*n* = 3 × 5). The experiment lasted for 3 days, during which the survival of the crayfish was recorded daily.

### 2.8. Absolute Quantification of WSSV

Genomic DNA was extracted from the samples 24 h after injection using the TIANamp Marine Animal DNA Kit (TianGen, Beijing, China), and the concentration was determined using a Nano-400A micro-spectrophotometer (Hangzhou Ansheng Instrument Co., Ltd., Hangzhou, China). Following previously reported methods, WSSV copy numbers were quantified using quantitative PCR (qPCR) based on the WSSV-specific primer *VP28*. Each reaction contained 200 ng of genomic DNA template. The PCR cycling conditions were as follows: a 5 min pre-denaturation at 95 °C, followed by 40 cycles, each consisting of 10 s of denaturation at 95 °C and 30 s of annealing at 55.5 °C. The primer sequences used in this study are listed in Table S1. Viral copy numbers were calculated based on a pre-prepared standard curve. The final results were expressed as the number of WSSV gene copies per nanogram of total DNA, and the inhibition rate was calculated using the following formula:

(1) Inhibition rate (%) = [(C−T)/C] × 100%

where C represents the viral copy number in the control group, and T represents the viral copy number in the experimental group.

### 2.9. Analysis of Related Gene Expression

Twenty-four hours after injection, total RNA was extracted from the samples using TRIzol total RNA extraction reagent (CWBIO, Taizhou, China), and cDNA was synthesized from the total RNA using the HiFiScript cDNA synthesis kit (CWBIO, Taizhou, China). All nucleic acid concentrations were determined using a micro-spectrophotometer. Using the obtained cDNA as a template, quantitative real-time PCR (qRT-PCR) was performed to detect the expression levels of immune-related genes. The total volume of each reaction was 15 μL, containing 7.5 μL of UltraSYBR Mixture (Low ROX) (CWBIO, Taizhou, China), 0.3 μL each of forward and reverse primers (10 μM; Sangon Biotech Co., Ltd., Shanghai, China), and 1 μL of cDNA template. The PCR cycling conditions were as follows: an initial denaturation at 95 °C for 5 min, followed by 40 cycles of denaturation at 95 °C for 10 s and annealing at 60 °C for 30 s. The primers used for qRT-PCR in this study are listed in Table S2. β-actin was used as the internal control. The relative fold change in WSSV gene expression was calculated using the 2^−ΔΔCt^ method.

### 2.10. Assay of Antioxidant Enzyme Activity

Twenty-four hours after injection, red crayfish were randomly selected from each group, and their gills, hepatopancreas, and intestinal tissues were collected. The tissues were placed in pre-chilled 0.9% saline solution to prepare tissue homogenates, which were then centrifuged at 4 °C and 4000 rpm for 10 min. The supernatant was collected for subsequent enzyme activity assays. The activities of superoxide dismutase (SOD) and catalase (CAT) were determined using commercial kits provided by the Nanjing Jiancheng Institute of Biological Engineering (Nanjing, China). The procedures were performed according to the kit instructions, and the final results were calculated using the following formula:

(2) $${\mathrm{SOD}}_{\mathrm{inhibition}\;\mathrm{rate}}\;\left(\%\right)\;=\;\left(1\;-\;\frac{\left(A3-A4\right)}{\left(A1-A2\right)}\right)\;\times \;100\%\;$$

(3) $$\mathrm{SOD}\;\mathrm{Activity}\;\left(U/\mathrm{mg}\;\mathrm{prot}\right)={\mathrm{SOD}}_{\mathrm{inhibition}\;\mathrm{rate}}\;÷\;50\%\;\times \;\frac{V_{\mathrm{Total}\;\mathrm{volume}\;}}{V_{\mathrm{Sample}}}\;\times \;N\;÷\;C_{\mathrm{pr}}$$

A1—control wells

A2—control blank wells

A3—test wells

A4—test blank wells

V_Total volume_—total volume of the reaction system, 0.24 mL

V_Sample_—sample amount, 0.02 mL

N—dilution factor prior to sample testing

C_pr_—protein concentration in the homogenized sample

(4) $$\mathrm{CAT}\;\mathrm{Activity}\;\left(U/\mathrm{mg}\;\mathrm{prot}\right)=\Delta A\;\times \;271\;÷\;V_{\mathrm{Sample}}\;÷\;T\;÷\;C_{\mathrm{pr}}$$

ΔA—A_Control_ − A_Test_

V_Sample_—sample size under this framework, 0.1 mL

T—60 s reaction time

C_pr_—protein concentration in the homogenized sample

### 2.11. Cytotoxicity Assessment

The cytotoxicity of CE SA nanoparticles was evaluated using the CCK-8 assay. Collect crayfish hemolymphocytes and mix them with pre-chilled anticoagulant (1:1) (anticoagulant: 27 mM Na_3_C_6_H_5_O_7_, 8 mM C_6_H_8_O_7_, 336 mM NaCl, 115 mM C_6_H_12_O_6_, dissolved in 1 L of deionized water, and sterilized by filtration through a 0.22 μm filter prior to use). Centrifuge at 1000× *g* for 5 min at 4 °C, discard the supernatant, and wash with phosphate-buffered saline (CPBS) (CPBS: 10 mM Na_2_HPO_4_, 10 mM KH_2_PO_4_, 150 mM NaCl, 10 μM CaCl_2_, 10 μM MnCl_2_, dissolved in 1 L deionized water, pH = 6.8), repeat the above procedure, resuspend the cell pellet in modified Leibovitz’s L-15 medium, then inoculate into sterile cell culture plates and culture at 25 °C. Cell toxicity was determined using a commercial assay kit provided by Shanghai GenScript (Shanghai, China). The procedure was performed according to the kit instructions. Finally, absorbance was measured at 450 nm using a microplate reader, and cell survival rates were calculated based on the changes in absorbance. All experiments were performed in triplicate.

### 2.12. Histopathological Analysis

Hematoxylin and eosin (H&E) staining was used to examine pathological changes in the hepatopancreas and intestinal tissues of crayfish. After harvesting the hepatopancreas and intestinal tissues, they were immediately fixed in a 4% glutaraldehyde solution. The fixed tissues were sequentially dehydrated using a gradient of 70%, 80%, 90%, 95%, and 100% ethanol, and after clearing with xylene, they were embedded in paraffin. The embedded tissues were sectioned into 4 μm thick sections. After deparaffinization with xylene and rehydration using a gradient of ethanol (100%, 95%, 90%, 80%, and 70%), the sections were stained with hematoxylin for 3 min, differentiated with 1% hydrochloric acid in ethanol, rinsed under running water until blue, and then stained with eosin for 5 min. Subsequently, the sections underwent dehydration, clearing, and mounting. The tissue sections were finally examined and photographed using a high-performance biological microscope (80I, Nikon, Tokyo, Japan).

### 2.13. Metabolomics Analysis

Intestinal tissues from the WSSV group and the CE SA group were selected for non-targeted metabolomics analysis; each group included three biological replicates (*n* = 3). Metabolites were detected using an HPLC–MS/MS platform. Raw data were processed using Progenesis QI v3.0 software (Waters Corporation, Milford, MA, USA) for peak detection, peak extraction, alignment, and integration, and metabolite annotation was performed using the HMDB and Metlin databases. After removing features with more than 20% missing values in each group, missing data were imputed with the minimum value, and total ion peak areas were normalized. Variables with an RSD > 30% in the QC samples were excluded, and a log10 transformation was performed to obtain the data matrix for analysis. PCA and OPLS-DA analyses were performed using the ropls package (Version 1.6.2) in R. Differentially expressed metabolites were identified using VIP > 1, *p* < 0.05, and fold change (FC) criteria. Metabolite annotation was based on the HMDB and KEGG databases, and pathway enrichment analysis was conducted using the Python 3.9 package scipy.stats.

### 2.14. Statistical Analysis

Statistical analyses were performed using SPSS 26.0 software (SPSS Inc., Chicago, IL, USA). Differences between groups were tested using one-way analysis of variance (ANOVA) and corrected with Dunnett’s multiple comparison test. Survival analysis was performed using the Kaplan–Meier method. All results are presented as the mean ± standard deviation (SD). Asterisks indicate statistically significant differences between experimental groups and the control group, with * *p* < 0.05 and ** *p* < 0.01.

## 3. Results

### 3.1. Characterization of CE SA Self-Assembled Nanoparticles

**Figure 1:**
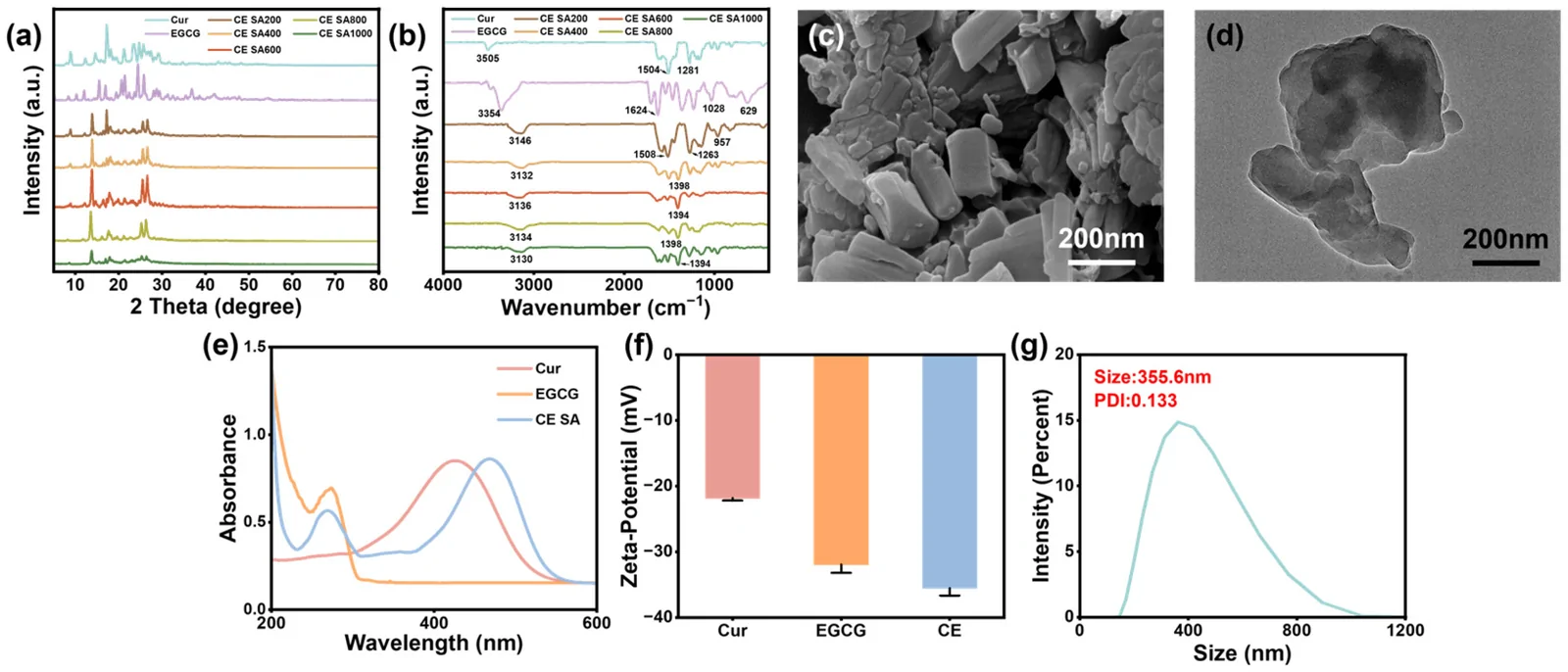
(**a**) XRD patterns of Cur, EGCG, and CE SA at different ratios; (**b**) FTIR spectra of Cur, EGCG, and CE SA at different ratios; (**c**) SEM image of CE SA; (**d**) TEM image of CE SA; (**e**) UV–vis absorption spectra of Cur, EGCG, and CE SA; (**f**) zeta potentials of Cur, EGCG, and CE SA; (**g**) particle size distribution and PDI results of CE SA.

To identify the optimal self-assembly ratio, structural characterization was performed on CE SA samples with different EGCG loadings. The XRD results are shown in Figure 1a. Cur exhibited multiple distinct and sharp diffraction peaks, indicating high crystallinity, while EGCG also displayed certain crystalline features. As the EGCG loading increased, the characteristic diffraction peaks in the CE SA samples gradually intensified. The CE SA 600 sample exhibited the highest peak intensity, with most characteristic peaks significantly enhanced, indicating a marked increase in the system’s crystallinity and the highest degree of self-assembly. When EGCG was further increased to 800 and 1000, the intensity of some diffraction peaks increased again, suggesting the reappearance of a certain degree of ordered structure in the system.

The FTIR results are shown in Figure 1b. Cur exhibits an O–H stretching vibration peak near 3505 cm^−1^ and a C=O vibration peak at 1624 cm^−1^; EGCG exhibits a broadened hydroxyl absorption peak near 3354 cm^−1^ and characteristic peaks related to aromatic rings and phenolic hydroxyl groups near 1504 cm^−1^ and 1281 cm^−1^. After self-assembly, the O–H characteristic peak in each CE SA sample shifted toward lower wavenumbers, while the aromatic ring-related vibrational peak near 1500 cm^−1^ and the phenolic hydroxyl-related peak near 1400 cm^−1^ shifted to varying degrees. The peak shift was most pronounced in the CE SA 600 sample, while the magnitude of the peak shift in the CE SA 800 and 1000 samples did not increase further compared to CE SA 600.

SEM results (Figure 1c) show that CE SA exhibits a distinct lamellar aggregation structure; TEM results (Figure 1d) further confirm the formation of a nanosheet structure with thin edges and a certain degree of transparency. UV–vis results (Figure 1e) show that CE SA retains the characteristic absorption peaks of both Cur and EGCG, and the Cur characteristic absorption peak exhibits a red shift. Zeta potential analysis results showed that the surface potential of CE SA was approximately −36 mV (Figure 1f), with an average particle size of approximately 355.6 nm and a PDI of 0.133 (Figure 1g), indicating that the resulting nanoparticles possess good uniformity and dispersion stability.

### 3.2. Stability of CE SA Under Different pH Conditions

**Figure 2:**
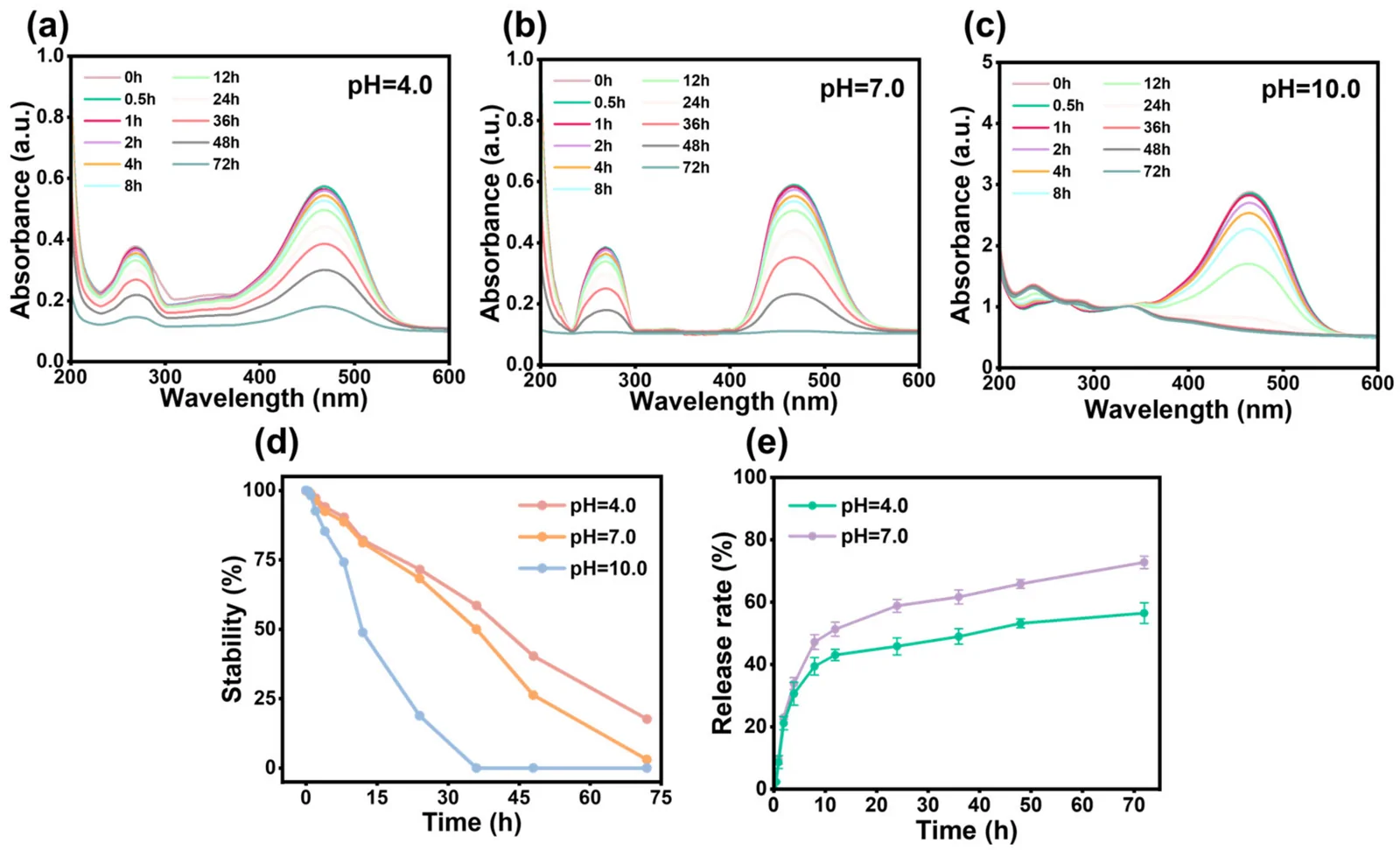
(**a**–**c**) Changes in the UV-vis spectra of CE SA after incubation for 0–72 h at pH 4.0, pH 7.0, and pH 10.0, respectively. (**d**) Stability of CE SA at different pH conditions (pH 4.0, pH 7.0, and pH 10.0). (**e**) In vitro cumulative release profiles of CE SA at different pH conditions (pH 4.0 and pH 7.0). Data are presented as mean ± SD (*n* = 5).

To evaluate the stability of CE SA under different conditions, continuous monitoring was conducted at pH 4.0, 7.0, and 10.0. As shown in Figure 2a–c, CE SA retained distinct characteristic absorption peaks under both acidic and neutral conditions. The peak at approximately 270 nm corresponds to the π→π* transition absorption peak generated by the aromatic ring structure of EGCG, while the peak at approximately 470 nm corresponds to the characteristic absorption peak of the Cur conjugated double bond system.

As time elapsed, the intensities of the absorption peaks in all groups gradually decreased, but the rates of decline showed significant differences. Under pH 4.0 conditions, the absorption peaks near 270 nm and 470 nm changed relatively slowly, retaining distinct absorption signals even after 72 h; under pH 7.0 conditions, the absorption peaks gradually weakened, but the overall magnitude of change was lower than that in the alkaline environment. In contrast, the absorption peak decay was most pronounced at pH 10.0, where the characteristic peak near 270 nm weakened significantly in the early stages, while the absorption peak near 470 nm declined rapidly within 24 h and had essentially disappeared by 36 h.

As shown in Figure 2d, the stability of CE SA continued to decline over time, but the rate of decline differed significantly under different pH conditions. The pH 4.0 group exhibited the highest stability, retaining approximately 18% after 72 h; the pH 7.0 group decreased to approximately 3% at 72 h; and the pH 10.0 group declined the fastest, losing stability almost entirely after 36 h. The results indicate that CE SA exhibits better structural stability in an acidic environment, whereas an alkaline environment significantly accelerates its degradation process.

### 3.3. EGCG Release Behavior of CE SA Under Different pH Conditions

As shown in Figure 2e, CE SA exhibited typical biphasic release characteristics under different pH conditions, with a relatively fast release rate during the initial phase (0–8 h), followed by a gradual transition to a slow-release phase. At pH 4.0, the cumulative release rate of EGCG reached approximately 42% at 8 h and then slowly increased to about 56% at 72 h, whereas at pH 7.0, the release rate was significantly higher than under acidic conditions, with the cumulative release rate reaching approximately 72% at 72 h. The results indicate that CE SA is capable of sustained release and exhibits certain pH-responsive release characteristics.

### 3.4. Effects of CE SA on WSSV Replication and Infection-Related Mortality

**Figure 3:**
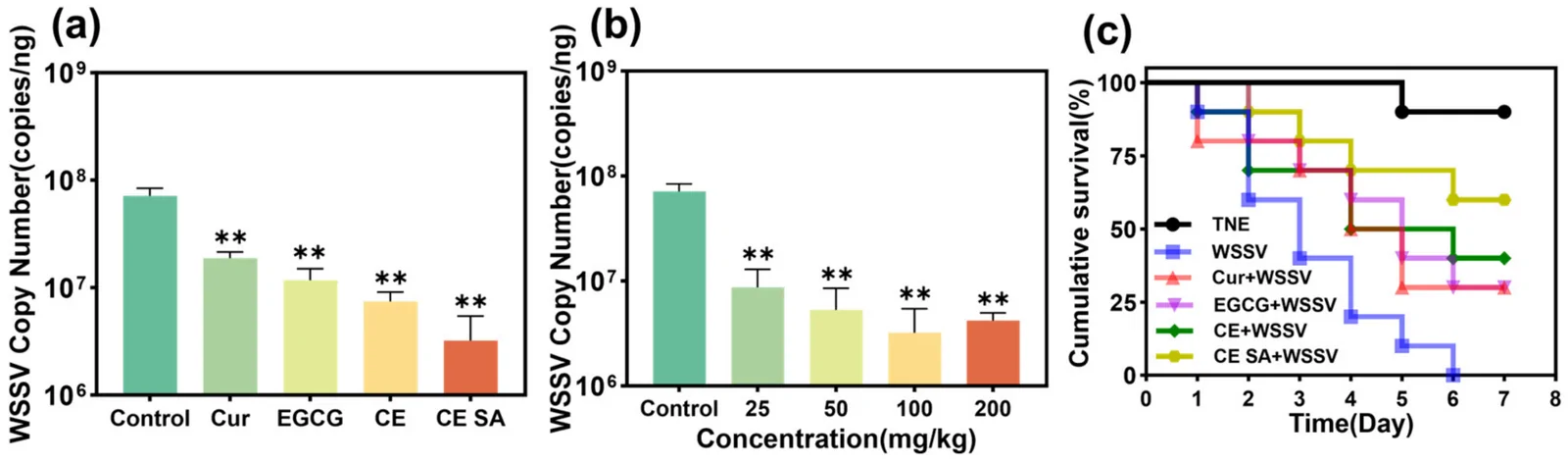
(**a**) WSSV copy numbers in *Procambarus clarkii* following injection with Cur, EGCG, CE, and CE SA. (**b**) Statistical results of WSSV copy numbers in crayfish following injection of different concentrations (25, 50, 100, and 200 mg/kg) of CE SA. Data are presented as mean ± SD (*n* = 5). An asterisk indicates a significant difference between a treatment group and the control group (** *p* < 0.01). (**c**) Cumulative survival rates of crayfish following injection with WSSV and drugs/WSSV. Each group included three replicate samples; survival rates were continuously monitored and recorded daily for 7 days for each replicate (*n* = 20).

Prior to conducting in vivo antiviral experiments, safety evaluations were performed on Cur, EGCG, CE, and CE SA at different concentrations (25, 50, 100, and 200 mg/kg). The results showed that none of the treatment groups caused crayfish mortality, indicating that these compounds are safe and suitable for use in subsequent in vivo and in vitro antiviral studies. As shown in Figure 3a, following WSSV infection, the viral copy number in the control group was approximately 7.0 × 10^6^ copies/ng. Compared with the control group, both Cur and EGCG treatments significantly reduced the viral load (*p* < 0.01), to approximately 1.8 × 10^7^ and 1.1 × 10^7^ copies/ng, respectively. The CE group further decreased to approximately 7.0 × 10^6^ copies/ng, while the CE SA group was only approximately 3.0 × 10^6^ copies/ng, the lowest among all treatment groups, representing a reduction of approximately 95.7% compared to the control group. Furthermore, results from different concentrations of CE SA treatment (Figure 3b) showed that viral copy numbers gradually decreased with increasing dose, reaching the lowest level at 100 mg/kg (approximately 3.0 × 10^6^ copies/ng); they then slightly rebounded after increasing to 200 mg/kg. The results indicate that 100 mg/kg is the optimal concentration for CE SA to inhibit WSSV replication.

As shown in Figure 3c, the survival rate in the TNE group remained above 90% throughout the experiment, whereas the WSSV group died rapidly after infection, with the survival rate dropping to 0% by day 6. All drug treatments improved crayfish survival rates to varying degrees. The final survival rates were approximately 30% in both the Cur and EGCG groups, 40% in the CE group, and 60% in the CE SA group—the highest among all treatment groups. The results indicate that CE SA can significantly reduce mortality caused by WSSV infection and enhance crayfish survival.

### 3.5. Effects of CE SA on WSSV and Related Gene Expression in Infected Crayfish

**Figure 4:**
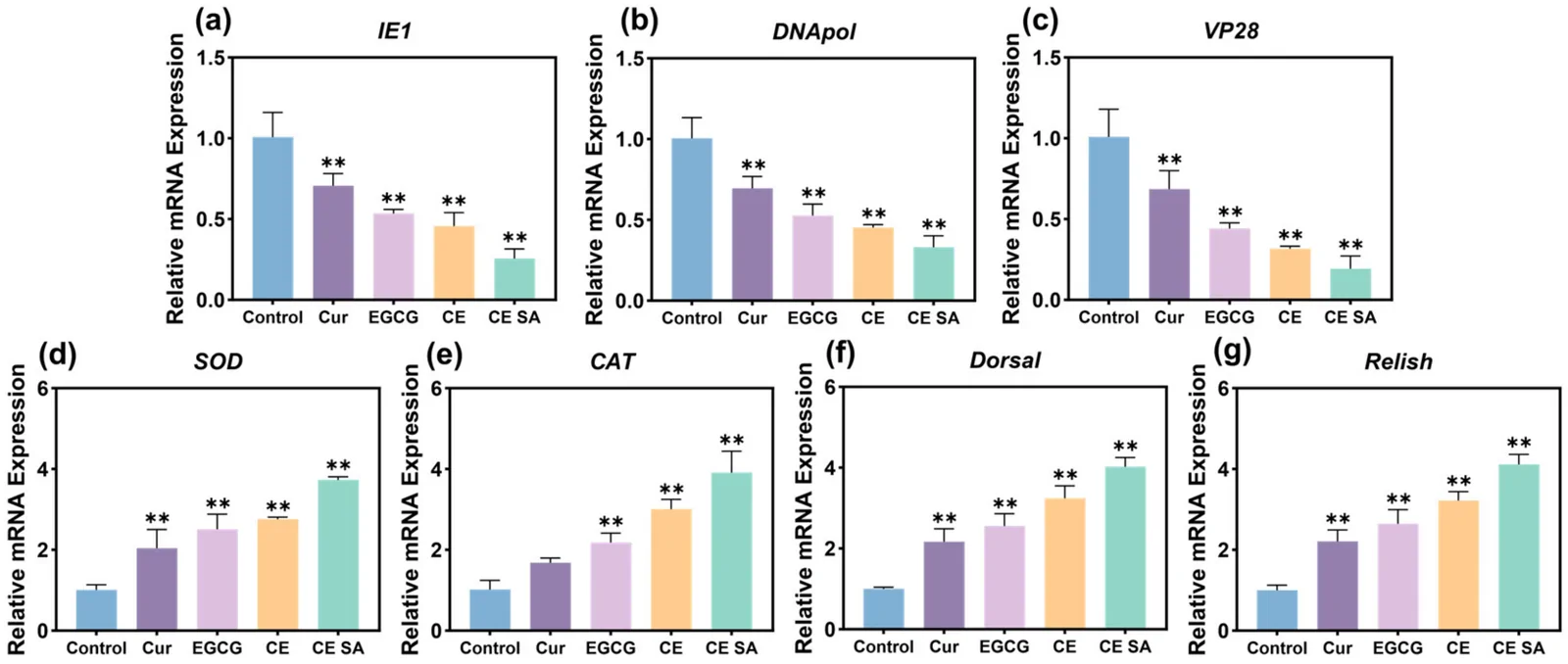
Relative expression levels of WSSV-related genes (**a**) *IE1*, (**b**) *DNApol*, and (**c**) *VP28* in crayfish, relative expression levels of antioxidant-related genes (**d**) *SOD* and (**e**) *CAT*, and immune-related genes (**f**) *Dorsal* and (**g**) *Relish* in crayfish following injection of Cur, EGCG, CE, and CE SA; β-actin was used as the internal control. Data are presented as mean ± SD (*n* = 5). An asterisk indicates a significant difference between the treatment group and the control group (** *p* < 0.01).

To evaluate the regulatory role of CE SA on host antiviral and immune responses following WSSV infection, the expression levels of WSSV-related genes (*IE1*, *DNApol*, and *VP28*) as well as immune and antioxidant-related genes (*SOD*, *CAT*, *Dorsal*, and *Relish*) were measured. As shown in Figure 4a–c, compared with the Control group, all treatment groups reduced the expression of WSSV-related genes to varying degrees, with the CE SA group exhibiting the most pronounced inhibitory effect. The expression level of the *IE1* gene in the CE SA group decreased to approximately 0.3 times that of the Control group, while the expression levels of *DNApol* and *VP28* decreased to approximately 0.35 times and 0.2 times, respectively, all significantly lower than those in the Cur, EGCG, and CE groups. In contrast to the downregulation of viral genes, immune and antioxidant-related genes showed an overall upregulation trend (Figure 4d–g). Specifically, the expression level of the *SOD* gene in the CE SA group was approximately 3.7 times that of the Control group, while *CAT* increased to approximately 3.9 times. The immune-related genes *Dorsal* and *Relish* were also significantly elevated, at approximately 4.0 and 4.1 times that of the Control group, respectively. Overall, CE SA treatment significantly suppressed the expression of WSSV-related genes while simultaneously enhancing the expression levels of host antioxidant and immune-related genes.

### 3.6. Effects of CE SA on Antioxidant Enzyme Activity in WSSV-Infected Crayfish

**Figure 5:**
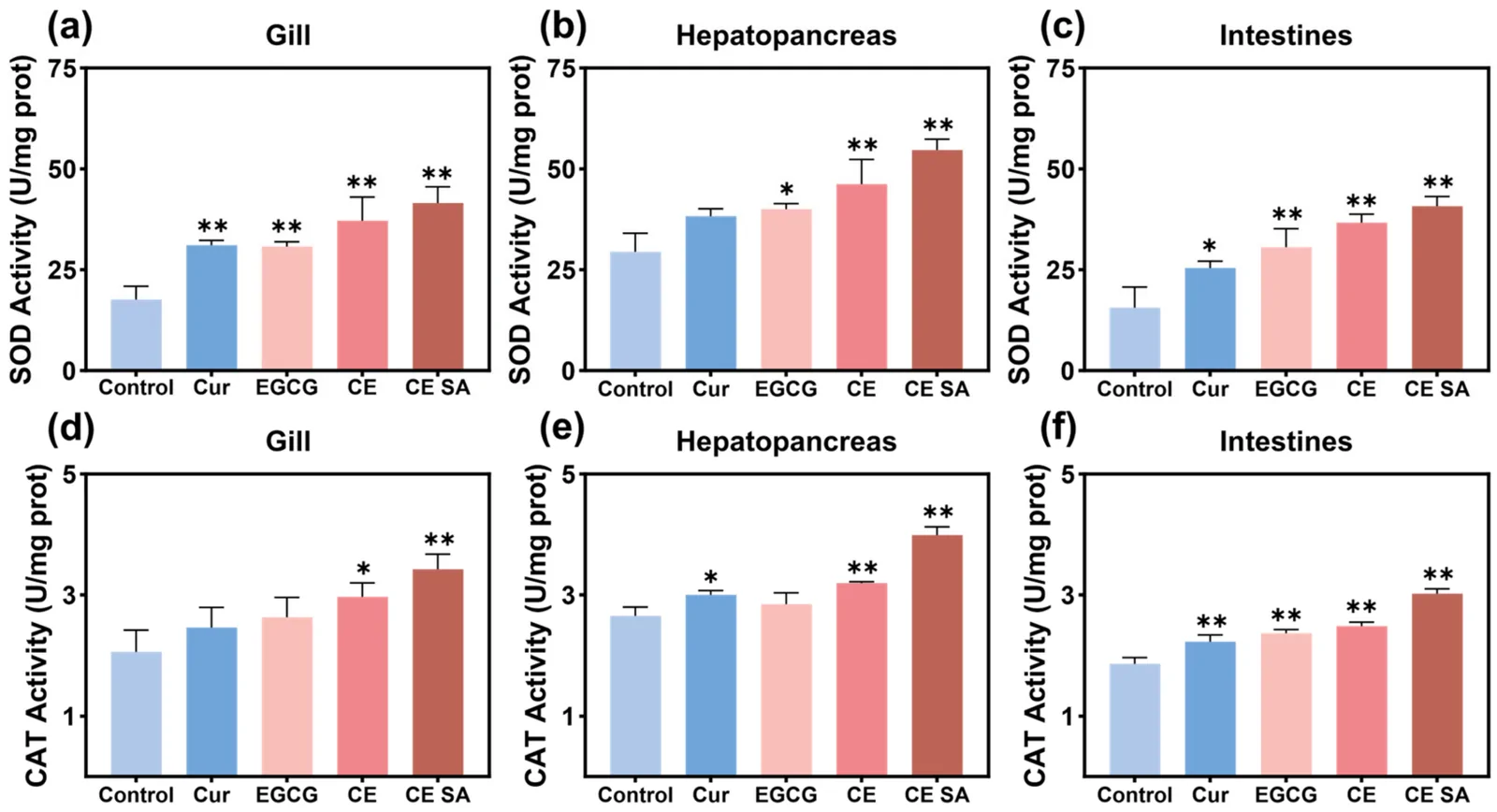
(**a**–**c**) SOD activity in the gills, hepatopancreas, and intestine; (**d**–**f**) CAT activity in the gills, hepatopancreas, and intestine. Data are presented as mean ± SD (*n* = 5). An asterisk indicates a significant difference between the treatment group and the control group (* *p* < 0.05, ** *p* < 0.01).

To evaluate the effects of different treatments on the antioxidant capacity of crayfish, SOD and CAT enzyme activity levels were measured in gill, hepatopancreas, and intestinal tissues. As shown in the figure (Figure 5a–c), regarding SOD activity, compared with the Control group, treatments with Cur, EGCG, CE, and CE SA all increased SOD activity in different tissues. In gill tissue, SOD activity in the CE SA group was approximately 2.4 times that of the Control group; in the hepatopancreas, the CE SA group had the highest SOD activity, approximately 1.9 times that of the Control group; a similar trend was observed in intestinal tissue, where SOD activity in the CE SA group was approximately 2.6 times that of the Control group, significantly higher than in the groups treated with individual active ingredients. The CAT activity results (Figure 5d–f) generally followed the same trend as SOD. In gill tissue, CAT activity in the CE SA group was approximately 1.6 times that of the Control group; in the hepatopancreas, the CE SA group showed the most significant increase in CAT activity, reaching approximately 1.5 times that of the Control group; in intestinal tissue, CAT activity in the CE SA group increased to approximately 1.6 times that of the Control group, significantly higher than in the other treatment groups. Overall, CE SA treatment demonstrated stronger antioxidant enzyme activation capacity across all tissues.

### 3.7. Cytotoxicity Assessment of CE SA

**Figure 6:**
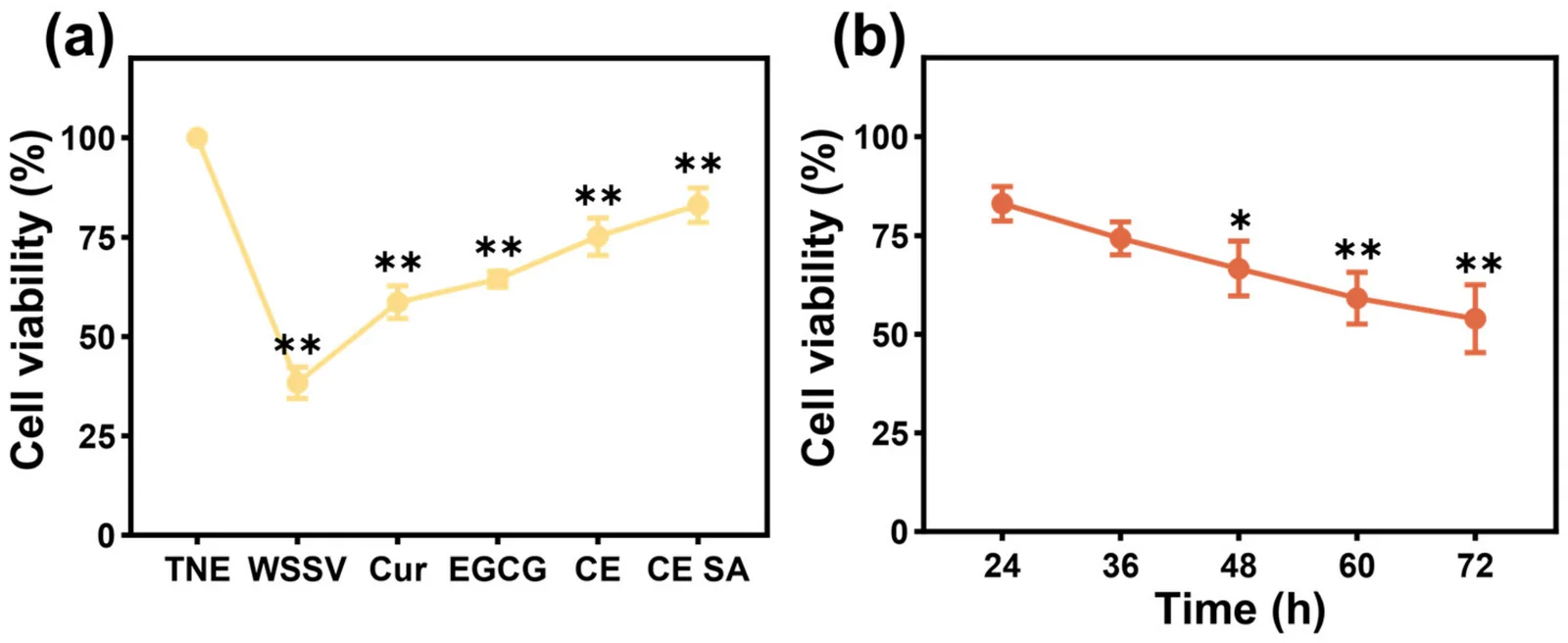
(**a**) Survival rates of crayfish hemolymphocytes under different treatment conditions (TNE, WSSV, Cur, EGCG, CE, and CE SA groups). (**b**) Survival rates of crayfish hemocytes during a 24 to 72 h culture period following CE SA injection. Data are presented as mean ± SD (*n* = 5). Asterisks indicate significant differences between groups (* *p* < 0.05, ** *p* < 0.01).

As shown in Figure 6, cell viability following WSSV infection decreased significantly from 100% in the TNE group to approximately 39%, indicating that the virus caused significant damage to the cells. After drug treatment, cell viability recovered to varying degrees: the Cur, EGCG, and CE groups showed cell viability of approximately 59%, 64%, and 75%, respectively, while the CE SA group reached approximately 83%, the highest among all treatment groups (Figure 6a). Furthermore, time-dependent experimental results (Figure 6b) showed that cell viability gradually decreased over time following CE SA treatment, dropping from approximately 83% at 24 h to approximately 54% at 72 h, but remained above 50% throughout the entire experimental period. The results indicate that CE SA can effectively alleviate WSSV-induced cellular damage while exhibiting good biocompatibility and low cytotoxicity.

### 3.8. The Effect of CE SA on Histopathological Changes in WSSV-Infected Crayfish Tissues

**Figure 7:**
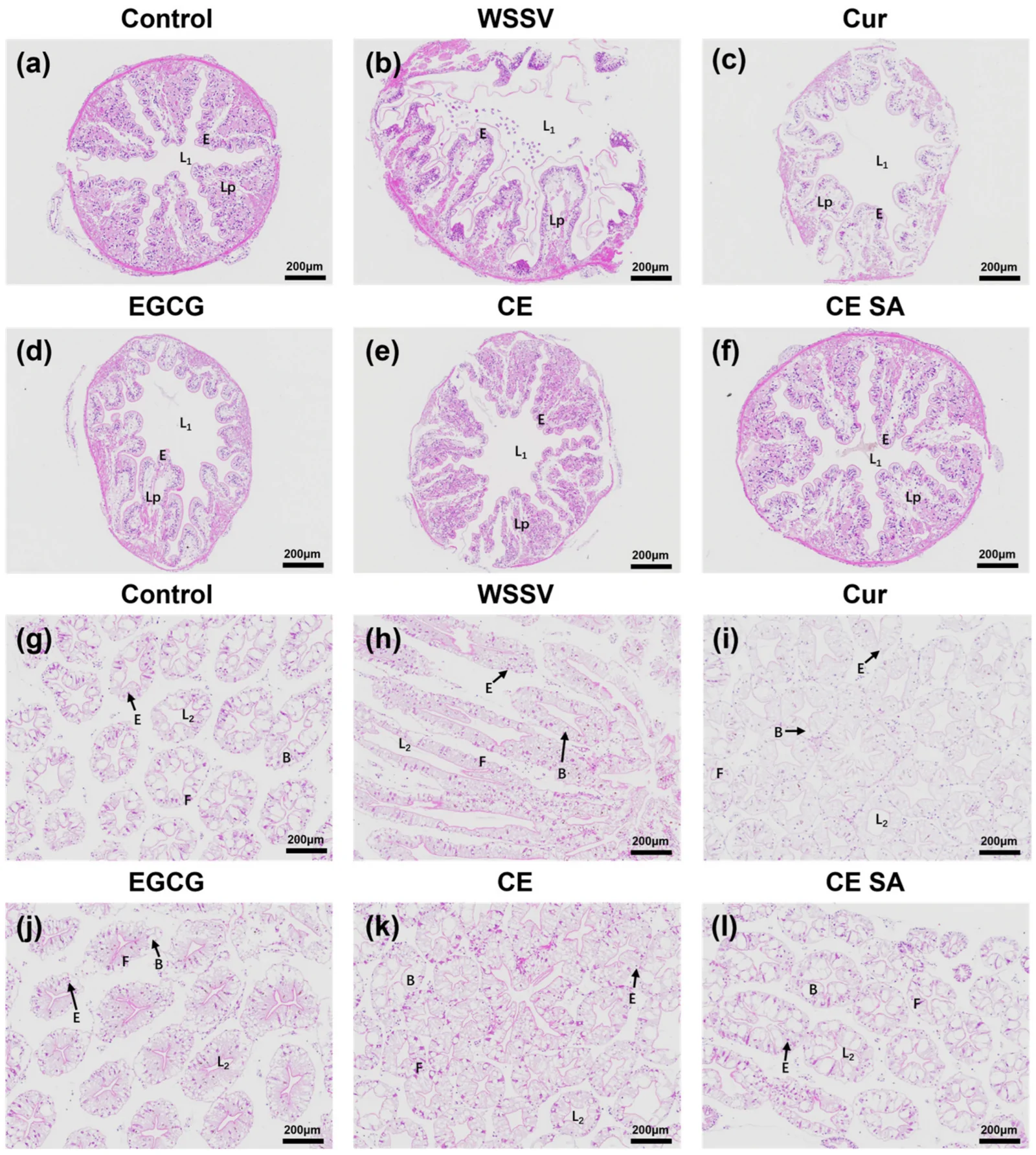
Histological observations were performed on paraffin sections of crayfish intestinal tissue (**a**–**f**) and hepatopancreatic tissue (**g**–**l**) following injection with Cur, EGCG, CE, and CE SA using H&E staining. L_1_ denotes the lumen; E denotes epithelium; Lp denotes the lamina propria; B denotes B cells; E denotes E cells; F denotes F cells; L_2_ denotes the inner lumen.

As shown in Figure 7a–f, the intestinal tissue in the Control group exhibited an intact overall structure, with clear lumen (L) and uniform distribution of the epithelium (E) and lamina propria (Lp). Following WSSV infection, the intestinal tissue displayed significant pathological damage, manifested as lumen dilation, disruption of the epithelial structure, and loosening of the lamina propria; localized tissue rupture was also observed in some areas. Compared to the WSSV group, intestinal tissue damage was alleviated following treatment with Cur and EGCG, though some degree of tissue loosening and structural incompleteness persisted. In the CE group, intestinal tissue structure recovered further, with luminal and epithelial structures tending toward integrity. The CE SA group showed the most pronounced recovery, exhibiting relatively intact overall intestinal tissue structure, with clear epithelial and lamina propria structures, and an overall morphology approaching that of the Control group.

The results of the hepatopancreatic tissue sections are shown in Figure 7g–l. In the Control group, the hepatopancreatic structure was intact, with clear, star-shaped luminal (L) boundaries, and B, E, and F cells were normally distributed and arranged in an orderly manner. The WSSV group exhibited obvious histological lesions, including dilated lumina, loose cellular arrangement, and partial destruction of cellular structures. Tissue damage was somewhat alleviated after treatment with Cur and EGCG, but localized structural abnormalities remained observable. The integrity of the hepatopancreatic tissue in the CE group improved further, while the CE SA group showed the best recovery, with relatively tight cellular arrangement, distribution of B cells, E cells, and F cells tending toward normal, and significantly reduced tissue damage [24].

### 3.9. CE SA Reshapes the Host Metabolic Profile Following WSSV Infection and Regulates Key Metabolic Pathways

**Figure 8:**
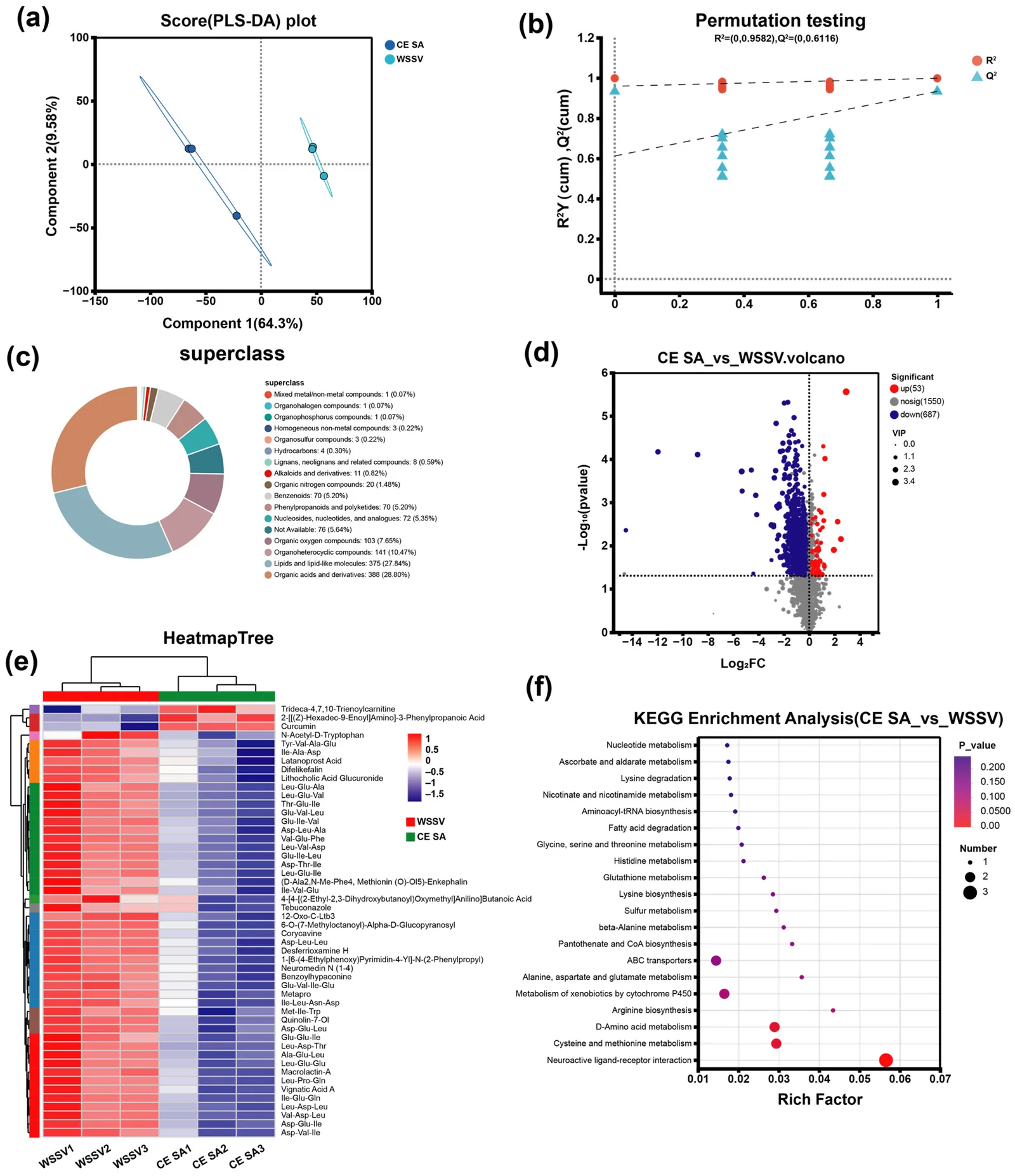
CE SA-regulated metabolic profiles and differential metabolite analysis in crayfish infected with WSSV. (**a**) PLS-DA score plots for samples from the WSSV and CE SA groups; (**b**) PLS-DA model exchange test (The horizontal axis represents the permutation retention level for the permutation test, while the vertical axis represents the values of R^2^ (red dots) and Q^2^ (blue triangles) for the permutation test. The two dashed lines represent the regression lines for R^2^ and Q^2^, respectively.); (**c**) HMDB classification statistics for differentially expressed metabolites; (**d**) volcano plot of differentially expressed metabolites, where red indicates significantly upregulated metabolites, blue indicates significantly downregulated metabolites, and gray indicates metabolites with no significant difference; (**e**) clustering heatmap of the top 50 differentially expressed metabolites; different colors indicate changes in relative metabolite abundance, with red representing high expression and blue representing low expression; (**f**) bubble plot of KEGG pathway enrichment analysis for differentially expressed metabolites; bubble size indicates the number of differentially expressed metabolites enriched in that pathway, and color indicates the significance of enrichment.

To investigate the regulatory role of CE SA on host metabolism following WSSV infection, non-targeted metabolomic analyses were performed on the WSSV group and the CE SA group. Partial Least Squares Discriminant Analysis (PLS-DA) results showed that samples from the two groups were clearly separated along the first and second principal components, with samples within each group clustering closely together, indicating that the overall metabolic profile of the host underwent significant changes following CE SA treatment (Figure 8a). The PLS-DA replacement test results indicated that the model did not exhibit overfitting and demonstrated good stability and predictive capability (Figure 8b). HMDB classification statistics showed that the differentially expressed metabolites were primarily classified as organic acids and their derivatives, lipids and lipid-like molecules, organic heterocyclic compounds, and organic oxides, suggesting that CE SA primarily regulates metabolites related to lipids, amino acids, and organic acids (Figure 8c). Volcano plot analysis revealed that, using VIP > 1 and *p* < 0.05 as screening criteria, a total of 740 differentially expressed metabolites were identified, of which 53 were upregulated and 687 were downregulated (Figure 8d). The clustering heatmap of the top 50 differentially expressed metabolites shows that the two sample groups can be clearly clustered into distinct branches, with differentially expressed metabolites exhibiting markedly different expression patterns between the two groups (Figure 8e). KEGG enrichment analysis results indicate that the differentially expressed metabolites are primarily enriched in metabolic pathways such as Neuroactive ligand-receptor interaction, Cysteine and methionine metabolism, D-Amino acid metabolism, and ABC transporters (Figure 8f).

**Figure 9:**
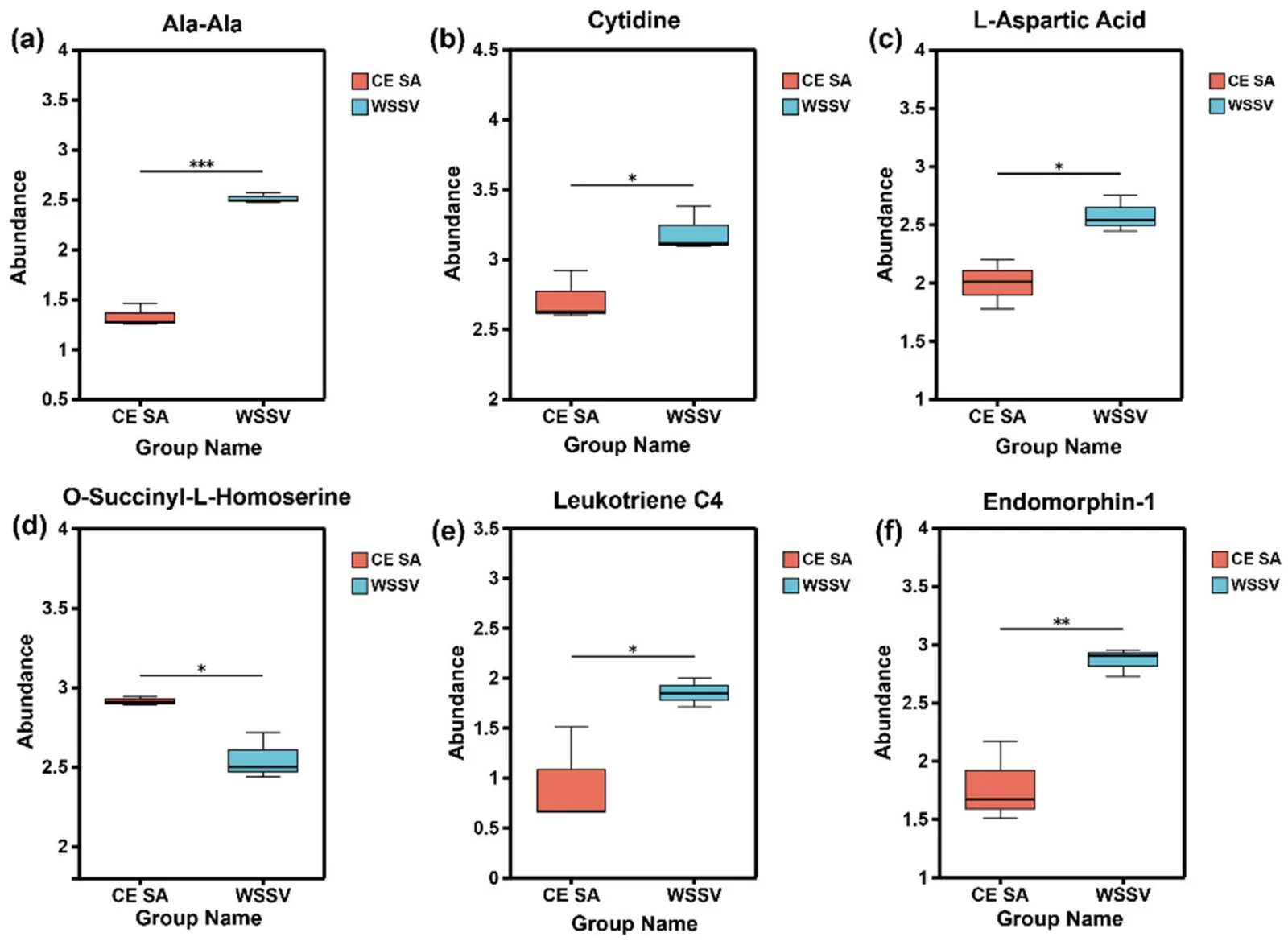
CE SA regulates changes in the relative abundance of metabolites associated with key metabolic pathways. (**a**) Ala-Ala; (**b**) Cytidine; (**c**) L-Aspartic Acid; (**d**) O-Succinyl-L-Homoserine; (**e**) Leukotriene C4; (**f**) Endomorphin-1. Data are expressed as mean ± standard deviation (Mean ± SD) (*n* = 3). Statistical analysis was performed using Student’s *t*-test (* *p* < 0.05, ** *p* < 0.01, *** *p* < 0.001).

Based on the KEGG enrichment analysis results, representative differentially expressed metabolites from the four major enriched pathways were further analyzed (Figure 9a–f). Compared with the WSSV group, the expression of Ala-Ala, cytidine, L-aspartic acid, leukotriene C4, and endomorphin-1 was significantly reduced after CE SA treatment, while the expression of O-succinyl-L-homoserine was significantly increased.

## 4. Discussion

### 4.1. Analysis of the Formation Mechanism and Optimal Composition of CE-SA Self-Assembled Nanoparticles

The XRD and FTIR results collectively confirm that effective molecular self-assembly has occurred between Cur and EGCG. As the EGCG ratio increased from 200 to 600, the XRD peaks gradually intensified, indicating the formation of a more ordered molecular arrangement within the system. Previous studies have shown that, under appropriate ratio conditions, polyphenolic natural products can form stable supramolecular structures through hydrogen bonding, π–π stacking, and hydrophobic interactions, thereby enhancing the order and stability of the system [25,26].

FTIR results further elucidated the intermolecular interaction mechanisms during the self-assembly process. Compared to the monomers, the O–H stretching peak in the CE SA shifted toward lower wavenumbers from 3354 cm^−1^, indicating the formation of strong hydrogen bonds between Cur and EGCG [27]. Concurrently, the aromatic ring skeletal vibration peak near 1504 cm^−1^ and the associated absorption peak near 1400 cm^−1^ shifted, indicating significant π–π stacking between aromatic rings accompanied by electron cloud redistribution [28,29]. Among these, the peak shift was most pronounced in the CE SA 600 group, suggesting the strongest intermolecular interactions at this ratio. It is worth noting that when the EGCG ratio was further increased to 800 and 1000, the intensity of the XRD peaks actually decreased, while the shifts in FTIR peak positions did not further increase. This suggests that an excess of EGCG did not continue to promote molecular assembly. On the contrary, an excess of EGCG may have altered the molecular packing scheme in the system, disrupting the original optimal supramolecular structure and thereby reducing the overall order [30]. Therefore, the appearance of the highest diffraction peak at the 600 ratio does not simply indicate increased crystallinity, but rather reflects that Cur and EGCG form the most stable molecular assembly structure at this ratio.

The lamellar nanostructures observed in the SEM and TEM results further confirm the formation of the molecular assembly. Meanwhile, the red shift in the characteristic Cur absorption peak in the UV–vis spectrum indicates electron coupling between aromatic rings [31], consistent with the π–π stacking revealed by FTIR. Furthermore, CE SA exhibited a high negative potential (−36 mV) and a low PDI value (0.133), indicating good colloidal stability and uniform dispersion [32,33]. Based on the combined XRD and FTIR results, the CE SA 600 group demonstrated the strongest intermolecular interactions and the highest structural order in this experiment, and was therefore identified as the optimal self-assembly ratio for subsequent experiments in this study. Hereinafter, whenever “CE SA” is mentioned, it refers to this specific ratio.

### 4.2. Analysis of the pH-Response Stability and Release Mechanism of CE SA

CE SA relies primarily on hydrogen bonding, π–π stacking, and hydrophobic interactions to form a stable structure; therefore, the pH of the environment has a significant impact on its stability. This study found that CE SA exhibits good stability under acidic conditions but rapidly destabilizes under alkaline conditions. Previous studies have shown that in acidic environments, the phenolic hydroxyl groups of Cur and EGCG primarily exist in a protonated form, which helps maintain hydrogen bond networks and hydrophobic interactions [34,35]. However, as pH increases, the deprotonation of phenolic hydroxyl groups leads to weakened hydrogen bonds and enhanced electrostatic repulsion, thereby promoting the dissociation of the self-assembled structure [36].

Notably, the absorption peak near 270 nm is significantly weakened or even disappears at pH 10, which is related to the susceptibility of EGCG to oxidation under alkaline conditions [37]. Studies have shown that the catechol structure of EGCG can be oxidized to form quinones and polymerization products, leading to the destruction of its characteristic conjugated structure and consequently causing the disappearance of the absorption peak near 270 nm [38,39]. Concurrently, the characteristic Cur peak near 470 nm also rapidly decays. This is attributed to the hydrolysis and oxidative degradation of Cur in an alkaline environment, which disrupts its conjugated structure [40]. Therefore, the attenuation of absorption peaks under alkaline conditions results from the combined effects of CE SA disassembly, EGCG oxidation, and Cur degradation.

Release experiments indicate that CE SA exhibits distinct sustained-release characteristics. The initial rapid release is primarily attributed to the diffusion of surface-adsorbed EGCG, while the subsequent slowing of the release rate is associated with the gradual release of active components from within the particles and the slow dissociation of the structure [41,42]. Although the release rate at pH 7.0 is higher than at pH 4.0, stability results show that CE SA is more stable under acidic conditions, suggesting that an acidic environment maintains the integrity of the self-assembled structure, thereby slowing the release of active components.

In summary, CE SA possesses both good acid stability and environment-responsive release capabilities. Its stability changes are not only related to the dissociation of the self-assembled structure but are also influenced by the alkaline degradation behavior of Cur and EGCG, providing an important foundation for its sustained anti-WSSV activity.

### 4.3. CE SA Enhances Resistance to WSSV by Inhibiting Viral Replication and Boosting the Host’s Immune Defenses

The results of this study indicate that both Cur and EGCG significantly reduce WSSV copy numbers, but CE SA exhibits a stronger inhibitory effect. Previous studies have shown that Cur exerts antiviral effects by regulating oxidative stress and interfering with viral replication processes [43], while EGCG inhibits viral infection and enhances the host’s antioxidant defense capacity [44]; both natural polyphenols possess good antiviral activity. Compared to individual components, CE SA demonstrated stronger antiviral activity, indicating that the self-assembly strategy can effectively enhance the utilization efficiency of the active ingredients. This may be attributed to the improved physicochemical stability and sustained-release capacity of the active ingredients provided by self-assembled nanostructures [45,46].

The results of viral gene expression further confirmed the inhibitory effect of CE SA on WSSV replication. *IE1*, *DNApol*, and *VP28* are involved in viral transcription regulation, DNA replication, and host infection, respectively, and their expression levels reflect viral replication activity. In this study, the expression of these genes decreased significantly following CE SA treatment, and the inhibitory effect was superior to that observed in the groups treated with Cur or EGCG alone, indicating that this system can effectively interfere with WSSV replication and proliferation.

In addition to directly inhibiting viral replication, CE SA also significantly enhanced the host’s immune and antioxidant defenses. Dorsal and Relish are key transcription factors in the Toll and IMD signaling pathways of crustaceans, and their activation is typically associated with enhanced innate immune responses [47]. In this study, the expression levels of both were significantly elevated, indicating that CE SA can activate the host immune system and enhance the body’s resistance to pathogen invasion. Concurrently, the expression of SOD and CAT genes, as well as the activity of their corresponding enzymes, were markedly increased, suggesting that CE SA can enhance the body’s ability to scavenge reactive oxygen species (ROS). Previous studies have shown that WSSV infection induces massive accumulation of ROS, leading to oxidative stress damage; conversely, enhancing the antioxidant defense system helps maintain the body’s redox balance and mitigate damage caused by viral infection [48,49]. It can thus be seen that CE SA not only inhibits WSSV replication but also enhances the crayfish’s resistance to WSSV by boosting the host’s immune defenses and antioxidant capacity.

Compared with traditional carrier-based nanodelivery systems, CE-SA employs a different assembly strategy. For example, polyacids and chitosan nanoparticles can enhance the stability and delivery efficiency of dsRNA through loading [50]. Unlike “carrier–active substance” systems, CE-SA uses Cur and EGCG as functional components that self-assemble into nanostructures via intermolecular interactions without the need for additional carriers, thereby improving the stability and utilization efficiency of the active ingredients and providing a new nano-assembly strategy for natural polyphenols against WSSV.

### 4.4. CE SA Alleviates WSSV-Induced Cellular and Tissue Damage

WSSV infection not only leads to massive viral replication but also induces oxidative stress and cellular damage, thereby impairing normal physiological functions. In this study, blood cell viability decreased significantly following WSSV infection, while treatment with Cur and EGCG improved cell viability to some extent, with CE SA showing the most pronounced effect. These findings are consistent with the observed reduction in viral copy numbers and increased antioxidant enzyme activity, suggesting that CE SA maintains normal cellular function by mitigating viral infection and oxidative stress-induced damage.

Histopathological observations further confirmed the protective effect of CE SA. Following WSSV infection, pathological changes such as dilated blood sinuses, epithelial damage, and loose tissue structure were observed in the gill tissue, while the hepatopancreas exhibited disorganized cell arrangement and tissue structural disruption, indicating that the viral infection had severely impaired the function of the host’s vital organs. In contrast, after CE SA treatment, tissue damage in the gills and hepatopancreas was significantly reduced, cell arrangement tended toward normality, and tissue structure was well restored. The alleviation of tissue damage is closely related to the suppression of viral replication and the enhancement of the host’s antioxidant defense capacity. Both Cur and EGCG possess the ability to regulate redox homeostasis and alleviate inflammatory damage [51,52], while the stable nanostructures formed through self-assembly further enhance the stability and sustained action of the active components within the body, thereby strengthening the tissue-protective effect [53]. The above results indicate that CE SA can further improve the host’s resistance to white spot syndrome virus infection by maintaining cell viability and protecting the functions of vital tissues and organs.

### 4.5. CE SA Maintains Host Metabolic Homeostasis and Enhances Resistance to WSSV by Regulating Key Metabolic Pathways

Viral infection can lead to metabolic reprogramming in the host, providing the energy and biosynthetic substrates required for viral replication [54,55]. Through non-targeted metabolomics analysis, this study found that the metabolic profile of WSSV-infected hosts underwent significant changes following CE SA treatment. Differentially expressed metabolites were primarily enriched in pathways such as neuroactive ligand-receptor interactions, cysteine and methionine metabolism, D-amino acid metabolism, and the ABC transport system, indicating that CE SA can effectively alleviate WSSV-induced metabolic dysregulation.

Following CE SA treatment, Endomorphin-1, Leukotriene C4, and L-aspartic acid in the neuroactive ligand-receptor interaction pathway were all significantly downregulated. Among these, Leukotriene C4 is an important inflammatory mediator, its excessive accumulation may promote inflammatory responses and tissue damage [56]. Endomorphin-1 is involved in neuro-immune interactions and the regulation of inflammation; changes in its levels may reflect alterations in the host’s neuroimmune status, suggesting that CE SA may contribute to maintaining host immune homeostasis by regulating the neuroimmune network [57]. L-aspartic acid, as a key intermediate in amino acid metabolism, is involved in multiple pathways, including cysteine and methionine metabolism as well as D-amino acid metabolism; its reduction may reflect CE SA’s regulatory effect on WSSV-induced abnormal amino acid metabolism [58,59]. Additionally, both Ala-Ala and cytidine showed a downward trend following CE SA treatment. Ala-Ala is involved in the amino acid cycle and protein metabolism; its changes may be related to metabolic remodeling under infection [60]. Cytidine is involved in nucleotide metabolism; its reduction may decrease the resources available for nucleic acid synthesis required for viral replication [61], consistent with the finding that CE SA inhibits WSSV replication. In contrast, levels of O-succinyl-L-homoserine increased; this metabolite is involved in sulfur-containing amino acid metabolism and may help maintain redox balance [62], consistent with the finding that CE SA enhances SOD and CAT activities. However, given that non-targeted metabolomics involves multiple comparisons of a large number of metabolites, this study was based on independent biological replicates and employed the Benjamini–Hochberg method to control the false discovery rate. Nevertheless, due to factors such as the number of biological replicates, individual variability, and the accuracy of metabolite identification, the identified differentially expressed metabolites and pathways still require further validation through targeted metabolomics.

In summary, CE SA can improve the metabolic imbalance caused by WSSV infection by regulating amino acid metabolism, inflammation-related metabolism, nucleotide metabolism, and redox processes, thereby enhancing the host’s antiviral defense capabilities. This study further elucidates the potential metabolic regulatory mechanisms underlying the antiviral activity of self-assembled nanoparticles derived from natural products against WSSV.

## 5. Conclusions

This study successfully developed a self-assembled nanoparticle (CE SA) composed of Cur and EGCG and systematically evaluated its protective effects on WSSV-infected crayfish. The results showed that CE SA exhibits good stability, biocompatibility, and sustained-release properties, and can effectively enhance the bioavailability of natural active ingredients. Compared with single components, CE SA significantly increased the survival rate of WSSV-infected shrimp, reduced viral replication levels, and enhanced the host’s immune response and antioxidant defense capacity. At the same time, CE SA effectively alleviated WSSV-induced damage to the hepatopancreas and intestinal tissues, improving the health status of host tissues. Furthermore, metabolomic analysis further elucidated the potential mechanisms underlying CE SA’s antiviral effects, revealing that CE SA regulates key metabolic processes such as amino acid metabolism, inflammation-related metabolism, nucleotide metabolism, and redox homeostasis, suggesting that metabolic remodeling may contribute to its protective effects against WSSV. The study results indicate that the self-assembly strategy not only overcomes issues such as the insufficient stability and limited in vivo duration of action of natural bioactive compounds but also enhances the host’s disease resistance by synergistically boosting antiviral, immunomodulatory, antioxidant, and metabolic regulatory functions. Overall, CE SA provides a simple, safe, and promising nanotechnology strategy for the efficient utilization of natural bioactive substances and offers new theoretical foundations and research directions for the development of green prevention and control technologies for viral diseases in aquatic animals.

## Data Availability

The original contributions presented in this study are included in the article/Supplementary Material. Metabolomic data have been deposited in the MetaboLights database under study accession (MTBLS15908). Further inquiries can be directed to the corresponding authors.

## Supplementary Materials

The following supporting information can be downloaded at https://www.mdpi.com/article/10.3390/antiox15101291/s1. Table S1: Primer sequences used in this study; Table S2: Primer sequences used in this study.
