# Supplementary material for: Self-Assembled Curcumin–Epigallocatechin Gallate Nanoparticles Enhance Antiviral Activity Against White Spot Syndrome Virus Through Immunomodulation, Antioxidant Effects, and Metabolic Regulation
Source: Antioxidants (Basel). 2026 Oct 5;15(10):1291. doi: 10.3390/antiox15101291 (PMC13701764; doi:10.3390/antiox15101291)
Supplement: Supplementary file 1 [file antioxidants-15-01291-s001.zip › antioxidants-4536171-supplementary.pdf]

Self-assembled curcumin-epicatechin gallate nanoparticles enhance antiviral activity against white spot syndrome virus through immunomodulation, antioxidant effects, and metabolic regulation

Songlin Luo[1], Xudong Yang[2], Shunqiang Wei[1], Chushun Yi[1], Fansheng Zeng[1], Fali Zhang[2], Wei Zhang[1], Xiuli Chen[3], Yongzhen Zhao[3], Yinghui Wang[1,2,*], Aiguo Huang[1,*]

1 Guangxi Laboratory on the Study of Coral Reefs in the South China Sea, Coral Reef Research Center of China, School of Marine Sciences, Guangxi University, Nanning 530004, China

2 Institute of Green and Low Carbon Technology, Guangxi Institute of Industrial Technology, Nanning 530201, China

3 Guangxi Academy of Fishery Sciences, Guangxi Key Laboratory of Aquatic Genetic Breeding and Healthy Aquaculture, Nanning 530021, China

* Corresponding authors

E-mail addresses: wyh@gxu.edu.cn (Yinghui Wang); aiguo_200891@126.com (Aiguo Huang)

Table S1 Primer Sequences Used in This Study

Primer	Primer sequence (5' to 3')
VP28-qF	AAACCTCCGCATTCCTGTGA
VP28-qR	TCCGCATCTTCTTCCTTCAT

Table S2 Primer Sequences Used in This Study

Primer	Primer sequence (5' to 3')
IE1-qF	GACTCTACAAATCTCTTTGCCA
IE1-qR	TGCTGATAAACTCTTGAAGGAA
DNApol-qF	CTCGCCAAAGTGAGTAGTGT
DNApol-qR	CCTTGTTGATGGAGGTAGAA
VP28-qF	AAACCTCCGCATTCCTGTGA
VP28-qR	TCCGCATCTTCTTCCTTCAT
CAT-qF	CGACCATACACCGCTTCAC
CAT-qR	TTTCAGGAATGCGTTCTCTATC
SOD-qF	GATCATCTTCTCATCTGACACCC
SOD-qR	CTCTGATTCTTCGTCCTCTGATG
Dorsal-qF	ATGGGATACCTCGACGTTATTC
Dorsal-qR	GCAGGAGGATAAGAATGCTGT
Relish-qF	GGTCATCAGTGATATTGAAGG
Relish-qR	TCTAATAAACGGAACCCAGAC
β-Actin-qF	ACCACTGCCGCCTCATCCTC
β-Actin-qR	CGGAACCTCTCGTTGCCAATGG
